# Editorial for the Special Issue on MEMS and Microfluidic Devices for Analytical Chemistry and Biosensing

**DOI:** 10.3390/mi13060896

**Published:** 2022-06-04

**Authors:** Stefano Zampolli

**Affiliations:** CNR-IMM Institute for Microelectronics and Microsystems, Italian National Research Council, Via P. Gobetti 101, 40129 Bologna, Italy; zampolli@bo.imm.cnr.it

The outbreak of the SARS-CoV-2 pandemic has made the general public aware of the breakthrough technologies which were developed in recent years for state-of-the-art biosensing, and terms such as clinical specificity and sensitivity are now widely understood. The need for reliable point-of-care diagnostic systems has never been felt as evidently as during the last few years. While PCR and other molecular diagnostic methods are now widely available in well-equipped medical structures, there is still need for the development of simpler, smaller, easy to use and lower cost diagnostic tools. Although less prominent, the same requirements apply for analytical-grade chemical sensing systems in environmental monitoring, safety and security.

Continuous developments in MEMS technology and microfluidics are key drivers for the miniaturization of lab-grade sensing systems. Micro-technologies and miniaturization allow for designing lightweight and small devices, but other advantages relevant though less obvious: reduced consumption of power and reagents, faster response times, increased sensitivity, reduced environmental footprint, availability of batch production processes for low-cost and disposable devices.

This special issue publishes 7 novel contributions in the fields of biosensing, lab-on-chip, organ-on-chip and related technologies such as numerical microfluidics studies, digital micro-fluidics and micromixers.

In [1], Yuan Tian et al. report on a novel MEMS microcantilever biosensor fabrication process which does not rely on expensive and potentially fragile SOI wafers. The novel design uses titanium as piezo-resistor between two thin Polyimide and SiO_2_ passive layers, featuring a comparatively low spring constant. The cantilevers are used in a Wheatstone bridge configuration and show a very low noise, and sensing experiments with functionalised cantilevers were performed. A minimum detectable ricin concentration of 10 ng/mL is reported, with a good linear behaviour over the 0–80 ng/mL concentration range.

The extremely low abundance of biomolecules in most sensing applications is the most well-known challenge. With the aim of reaching ever lower bio-detection limits, Yan Chen et al. [2] report on the development of an acoustic concentrator, named “acoustic tweezers”, which uses high frequency longitudinal acoustic waves to promote the concentration of the target molecules in the detection area of an electrolyte-gated graphene field effect transistor. The effect of the acoustic tweezers was validated under the microscope by IgG labelled with green fluorescence, and by comparison of the FET response (Dirac point shift) with and without acoustic accumulation.

To reach the goal of developing lower-cost and easy to use point-of-care analysis systems, Qin Huang et al. [3] propose a novel microfluidic lab-on-chip for isothermal parallel detection of multiple targets. The approach is based on a two-stage recombinase polymerase amplification and subsequent loop-mediated isothermal amplification (RPA + LAMP) microfluidic platform, which is demonstrated in a disc-shaped milled PMMA lab-on-chip. The system is able to detect different types of nucleic acid targets, e.g., bacteria and RNA viruses, at the same time. The detection is comparatively fast and operates at two isothermal setpoints, with high sensitivity and specificity. Disc rotation at up to 2000 rpm is used for priming and sample transfer. 

Digital microfluidics (DMF) are a very efficient technology to dispense, move, mix and transport micro-droplets of sample, reagents and reaction products. Electro-wetting on dielectric (EWOD) DMF are proposed by Zhen Gu et al. [4] for colorimetric sensing based on gold nanoparticles. The authors use a PCB-based DMF chip with a PTFE film as dielectric and hydrophobic layer and 0.1 mm electrode gaps. The contact angle of the gold nanoparticle solution was compared with pure water, resulting in a higher voltage required to move the suspension of nanoparticles. Evaporation effects were studied by UV/Vis absorbance measurements, and no significant evaporation was found after a 0.6 m travel distance. Colorimetric sensing tests were also performed targeting Hg^2+^, using 2 LED sources and 2 light detectors, and demonstrated a detection limit of 0.01 μMol.

In addition to the biosensors reported above, another increasingly prominent application of microfluidics is in the area of organ-on-chip devices. Larry J. Millet et al. [5] report on a platform to study radiation damage to pulmonary lumens, with the specific target of identifying candidate biomarkers of radiation damage. In this very interesting and novel pilot study a PDMS microfluidic platform is fabricated by molding from a Si master and loaded with HMVEC-L cell cultures to assess radiation damage after exposure to 10 Gy of Co-60 radiation. The original design of the first device was optimized by adding a third cell seeding port, to increase the cell loading uniformity. The perfusates of the cell cultures exposed to gamma irradiation were studied by LC-MS/MS and data searched against human proteome database, finding 26 statistically significant proteins that change in abundance between irradiated and nonirradiated tissue chip platforms.

While MEMS and microfluidics enable the miniaturization of the core sensing systems, in-field deployment often requires the miniaturization of auxiliary devices as well. In analytical chemical gas sensing applications, periodic recalibration is necessary, and in some cases the very low flow requirements and the high sensitivity of the sensors require novel approaches for an efficient calibration gas generation. Florian Noël et al. [6] present a low concentration gas mixture generator for the in-field calibration of a BTEX GC detector, which solves the problem of unnecessary consumption of both dilution and calibrated gases. The reported device is the experimental validation of a design studied by CFD in a previous paper. It uses a time-pulse approach and a micro-mixer device milled into PMMA, reaching dilutions up to 1:1000 with good precision and reproducibility at a low total gas flow of 25 NmL/min.

Phenomena at the microscale are sometimes difficult to observe, and novel techniques to characterize flow velocities in microchannels include the photobleaching effect. In an in-depth numerical study, Yu Chen et al. [7] use a model based on the convection–diffusion reaction equation to study the photobleaching process with reference to a gaussian laser focus region. The study allowed for the determination of the profiles of effective dye concentration and fluorescence, and to find the relationship between the photobleaching time constant obtained by experiments and the photochemical reaction coefficient.

I want to thank all the authors and teams who have contributed to the papers for this first volume of the Micromachines Special Issue on MEMS and Microfluidic Devices for Analytical Chemistry and Biosensing. 

A special thank goes also to the academic editors Katsuo Kurabayashi and Aiqun Liu for their precious contribution, and to Jerry Chen and Min Su of the Micromachines Editorial Office for their great support.
